# Toothbrushes may convey bacteria to the cystic fibrosis lower airways

**DOI:** 10.1080/20002297.2019.1647036

**Published:** 2019-08-07

**Authors:** Rebeca Passarelli Mantovani, Angela Sandri, Marzia Boaretti, Alessandra Grilli, Sonia Volpi, Paola Melotti, Gloria Burlacchini, Maria M. Lleò, Caterina Signoretto

**Affiliations:** a Department of Diagnostics and Public Health, Microbiology Section, University of Verona, Verona, Italy; b Cystic Fibrosis Center, Azienda Ospedaliera Universitaria Integrata (AOUI), Verona, Italy

**Keywords:** Bacterial oral reservoirs, oral hygiene, saliva, sputum, toothbrush care, cystic fibrosis

## Abstract

Recent findings indicate that the oral cavity acts as a bacterial reservoir and might contribute to the transmission of bacteria to the lower airways. Control of a potentially pathogenic microbiota might contribute to prevent the establishment of chronic infection in cystic fibrosis. We evaluated the presence of CF microorganisms in saliva and toothbrushes of CF patients and verify their possible transmission to lower airways.

**Methods**: We assessed the presence of *P. aeruginosa, S. aureus, S. maltophilia, A. xylosoxidans, S. marcescens*, and yeasts in saliva, toothbrushes and sputum of 38 CF patients and assessed the clonal identity of the strains occurring contemporary in multiple sites by PFGE.

**Results**: At least one of the investigated species was isolated from 60 saliva samples and 23 toothbrushes. *S. aureus* was the most abundant species, followed by Candida spp. 31 patients contemporary had the same species in sputum and saliva/toothbrush: in most cases, clonal identity of the strains among the different sites was confirmed.

**Conclusion**: Toothbrushes may be sources of oral contamination and might act as reservoirs favoring transmission of potentially pathogenic microorganisms from the environment to the oral cavity and eventually to the LAW. Oral hygiene and toothbrush care are important strategies to prevent CF lung infections.

Cystic fibrosis (CF) is the most common life-threatening recessively inherited disease in the Caucasian population, counting seventy thousand diagnosed people in the world [1]. The disease is caused by mutations in the *CFTR* (*CF transmembrane conductance regulator*) gene, situated in the long arm of chromosome 7 (7q31.2) and encoding the CFTR protein which mainly functions as a chloride channel in exocrine epithelia [2,3]. Up to date, over 2,000 different CFTR mutations have been identified (www.genet.sickkids.on.ca) causing CFTR dysfunction through a variety of different mechanisms. CFTR absence or low-functioning leads to insufficient chloride secretion in the secretory epithelia causing excessive reabsorption of ions and water and final dehydration and thickening of secretions. Pulmonary involvement is the major cause of CF morbidity and ultimate mortality. Thickened mucus obstructs distal airways promoting bronchiectasis and bacterial infections followed by intense neutrophil recruitment. Chronic infections and progressive inflammation favor tissue degradation leading to final lung failure [4].

Lung colonization with *Pseudomonas aeruginosa*, the most common bacterial species isolated from CF airways, is particularly difficult to eradicate and is associated with accelerated decline in lung function and poorer prognosis [5]. Other bacterial species play a role at different stages of the lung disease: *Staphylococcus aureus* and *Haemophilus influenzae* are the main pediatric pathogens while the *Burkholderia cepacia* complex (Bcc), *Achromobacter xylosoxidans, Stenotrophomonas maltophilia* and nontuberculous mycobacteria are mainly found in adults [6]. Many other microorganisms are also isolated from the sputum of CF patients but their clinical role is not clear yet; e.g. *Candida albicans* is a common commensal in the oropharynx and upper respiratory tract but chronic colonization of CF lower airways has been associated with worsening of lung function and greater rate of pulmonary exacerbations [7,8].

Microorganisms can colonize the lower airways by various routes. To reach the lungs, bacteria cross different anatomical areas, like the nose and paranasal sinuses on one hand and the oral cavity on the other, converging in the oropharynx. Also, oral bacteria can be released from dental plaque into the oral fluids which are then aspirated into the lower respiratory tract [9]. Depending on the state of health, the oral microbiota can also change; e.g. *S. aureus* and *P. aeruginosa* become predominant and constitute a more aggressive microbiota in critically ill patients [10]. In addition, the use of antibiotics can induce modifications in the oral microbiota and favor oral colonization with other bacterial species [11]. Recent studies investigated the role of the oral cavity as a possible source of bacteria for CF patients [12]. Bensel and colleagues found *P. aeruginosa, S. aureus* and *B. cepacia* in both periodontal pockets and sputum of the same patients [13]. In previous studies, *P. aeruginosa* was isolated also from the dorsum of the tongue, oral mucosa and saliva [14,15]. These observations suggest that the oral site could represent a reservoir of potentially pathogenic bacteria contributing to bacteria translocation and colonization of the lower airways. In this scenario, contaminated toothbrushes might contribute to the dissemination of microorganisms within the oral cavity of the same person or between different individuals [16] and subsequently to the airways, increasing the risk of respiratory infections. Indeed, they can get easily contaminated from various sources including the environment, hands and aerosols and can serve as reservoirs of potentially pathogenic microorganisms [17–20]. Toothbrushes can also become contaminated by the oral cavity itself. Retention and survival of bacteria on toothbrushes after brushing represent a possible cause of re-contamination of the mouth. Several studies [17,21] have shown that prolonged use of the toothbrush facilitates contamination by various microorganisms such as species of *Streptococcus, Staphylococcus, Lactobacillus, Pseudomonas, Klebsiella* and *Candida*, and by *Escherichia coli*. In CF children, *P. aeruginosa* and *S. aureus* nasopharyngeal carriage is up to 14%. In a pilot study, it has been found that *P. aeruginosa* and *S. aureus* are present on 15% and 22% of CF patients’ toothbrushes, respectively [17]. The actual impact of toothbrush contamination on bronchial primary colonization by *S. aureus* or *P. aeruginosa* seems to be very difficult to assess because bronchial colonization can occur from different sources and is highly dependent on the child’s pulmonary status. Currently, CF patients should follow the general recommendations of the health authorities including the Centers for Disease Control and Prevention, suggesting patients not to share toothbrushes, rinse their toothbrush thoroughly with tap water following brushing, not routinely cover the toothbrush or store it in closed containers allowing it to air-dry, and renew the toothbrush every 3 or 4 months, sooner if the bristles appear worn or splayed (www.cdc.gov/oralhealth/infectioncontrol/questions/toothbrush-handling.html).

In this study we aimed at evaluating the presence of classical CF bacterial species, namely *P. aeruginosa* and *S. aureus*, as well as other potentially pathogenic microorganisms recently isolated more and more frequently such as *S. maltophilia, A. xylosoxidans, Serratia marcescens* and *Candida* spp., in saliva, toothbrushes and sputum of CF patients. To verify the possibility of transmission between mouth/toothbrush and lower airways, we assessed the clonal identity of the strains present contemporary in both sites by Pulsed-Field Gel Electrophoresis (PFGE) [22,23]

## Materials and methods

### Patients

Thirty-eight patients followed at the Cystic Fibrosis Centre of Verona (Italy) were recruited to this study. The group included 13 adults, aged 18 to 26 years, and 25 pediatric patients, aged 7 to 17 years, diagnosed as never or intermittently colonized by *P. aeruginosa*. Informed consent was obtained as stated in projects CRCFC-CEPPO026 and CRCFC-CEPPO031 authorized by the local Ethical Committee. At routine visits, approximately every 3 months, sputum and saliva samples were collected and the used (2–3 months) toothbrushes were recovered.

### Routine visits and sampling

At every visit, a new toothbrush with soft bristles, along with a commercial toothpaste containing sodium fluoride, was given to the patients, together with a written protocol explaining how to use and store toothbrushes at home. During the visit, a dental hygienist also explained to the CF patient (and the parents, in case of pediatric patients) to rinse the toothbrush only with water, not to treat it with disinfectants, and to store it without cap and with the head turned upward. During the control visits, the used toothbrushes were recovered, placed in disposable plastic bags and immediately transported to the laboratory.

Sputum and saliva were also recovered from each enrolled patient. The saliva was recovered by spitting in a sterile tube, while the sputum was collected by natural expectoration or by a tracheal cannula when the patient was unable to expectorate.

### Bacterial strains isolation and identification

The toothbrush’s head was detached and underwent five cycles of sonication (30 sec each) in 5 ml BHI (Brain Heart Infusion) medium before incubation for 24 h at 37°C with shaking. Saliva and sputum samples were treated with 0.5–1 ml Sputolysin (Calbiochem) and incubated for 40 min at 37°C with shaking. Each sample (100 µl) was plated on Columbia blood (5% sheep blood), McConkey, Mannitol Salt and Sabouraud agar plates (BD Difco), and incubated at 37°C for 48 h. All plates were kept at RT for five additional days to recover small variant colonies and slow-growing strains. Isolated colonies were identified using MALDI-TOF-MS [24] and/or Vitek2 (Biomerieux). Isolates were stored in Microbanks (ProLab Diagnostics) at – 80°C.

### Pulsed-Field Gel Electrophoresis (PFGE)

Suspensions were prepared from individual colonies after culture on Blood Columbia agar. OD at 600 nm was measured and suspensions were diluted to 10^9^ CFU/ml in EDTA-saline buffer (75 mmol/L NaCl and 25 mmol/L EDTA, pH 7.5), then mixed with an equal volume of 1% low-melting-point agarose and allowed to solidify in a 100 μL plug mould. The agarose plug was incubated for 24 h at 37°C in 500 μL lysis buffer (6 mmol/L Tris–HCl (pH 7.6), 0.1 mol/L EDTA, 1 mol/L NaCl, 0.5% Brij®58 (polyoxyethylene (20) cetyl ether; Sigma), 0.4% sodium deoxycholate, 0.5% sodium lauryl sarcosine and 1 mg/mL lysozyme (and 10 µg/mL lysostaphin to *S. aureus*)). The lysis buffer was replaced with 500 μL proteinase K buffer (1% sodium lauryl sarcosine, 0.5 mol/L EDTA (pH 9) and proteinase K (50 μg/mL; Sigma)) and this solution was incubated with gentle shaking at 50°C for 20 h. The plugs were then washed four times for 30 min at 37°C with 10 mL of Tris–EDTA buffer (10 mmol/L Tris–HCl (pH 8) and 1 mmol/L EDTA). One-third of a slice of each plug was cut and incubated for 18–20 h with 30 U of SpeI, or SmaI, XbaI and Cfr9I in the restriction buffer (Thermo scientific). DNA restriction fragments were separated in a PFGE apparatus at 14°C, 6 V/cm, for a specific time for each species (19–23 h). The gel was stained with gel-red and visualized with a UV system.

### Genotype analysis

DNA band patterns were analyzed with InfoQuest FP software version 5.1 (Bio-Rad Laboratories, Hercules, USA), using the Dice correlation coefficient and Unweighted Pair Group Method with Arithmetic Mean (position tolerance and optimization between 1.0 and 1.5%). By applying the criteria of Tenover and colleagues [25], based on the differences in the numbers of bands, we identified a cut-off value of 80% similarity to correctly cluster the PFGE profiles. Consequently, the strains showing ≥80% similarity were considered to be a single clone.

## Results

Thirty-eight patients were included in this study, divided into two groups: 13 adults and 25 pediatrics (Table 1). During the study, induced sputum and saliva samples were collected and used toothbrushes were recovered, for a total of 74 saliva samples, 82 toothbrushes and 123 sputa (the number of sputa is higher than the other samples due to additional routine visits).

**Table 1.** Demographics data of patients included in the study.

	Study patients	
Group	Adults	Pediatrics
Number of patients	13	25
Average age (in years)	22 (18–26)	12 (7–17)
Male	8 (61.5%)	8 (32%)
Female	5 (38.5%)	17 (68%)

### Prevalence of the investigated bacterial species in saliva and toothbrush

**Figure 1.**
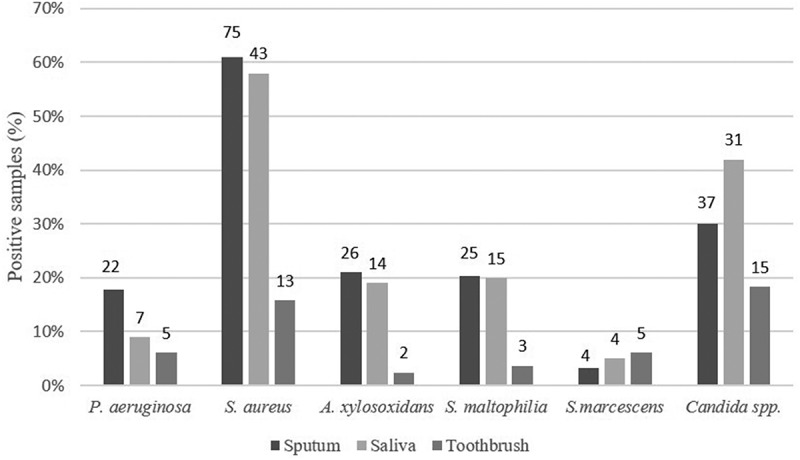
Prevalence of the investigated species of *P. aeruginosa, S. aureus, S. maltophilia, A. xylosoxidans, S. marcescens,* and *Candida* isolated from sputum, saliva and toothbrushes. Percentage of positive samples is indicated on the X-axis; number of positive samples is shown above bars.

At least one of the investigated species (*S. aureus, P. aeruginosa, S. maltophilia*, A. *xylosoxidans, S. marcescens*) was isolated from 60 saliva samples and from 23 toothbrushes. We observed that *S. aureus* was the most abundant species in saliva, outdistancing the prevalence of the other investigated species. Indeed, the number of saliva samples presenting *S. aureus* (58%) almost tripled the number of samples presenting any other species: *S. maltophilia* (20%), *A. xylosoxidans* (19%), *P. aeruginosa* (9%) and *S. marcescens* (5%). *S. aureus* was also the most abundant species collected from toothbrushes (16%) while the other investigated species were found in about 6% of these samples. Interestingly, among other isolated species, we observed a high incidence of *Candida* spp., which was isolated from 42% of saliva samples and 18% of toothbrushes. The overall prevalence of each species among all samples is reported in Figure 1.

### Contemporary presence of the same bacterial species in saliva/toothbrush and sputum

To investigate the possibility of transmission between the oral environment and lower airways, we searched for the contemporary presence of the investigated species in sputum and saliva/toothbrush. At least one of the investigated species was contemporary present in sputum and saliva of 31 patients; among these, 10 also presented with a positive toothbrush in the same period. Although these data suggested the occurrence of bacteria transmission between airways and mouth/toothbrush, we must also consider that during the expectoration procedure cross-contamination between sputum and saliva might happen. Thus, to be more restrictive and decrease the probability of false positives, we further analyzed only patients that contemporary had the same species in sputum and saliva/toothbrush at least twice during the study: with these parameters, 22 patients were contemporary positive in sputum and saliva; among these, two patients also presented two positive toothbrushes. Details on the bacterial species isolated from each of these patients are reported in Table 2.

**Table 2.** Bacterial species isolated from each patient that were contemporary positive in sputum and saliva/toothbrush twice (n = 19) or thrice (n = 3) during the study. Patients were adult (A) or pediatric (P). SAU = *S. aureus*, PAE = *P. aeruginosa*, AXY = *A. xylosoxidans*, PMA = *S. maltophilia*, SMA = *S. marcescens*, CAL = *Candida* spp. * = species isolated once in a type of sample.

	Sputum		Saliva		Toothbrush	
Patient	1st	2nd	1st	2nd	1st	2nd
A3	SAU	SAU	SAU	SAU	-	-
A5	AXY	AXY	AXY	AXY	-	-
A7	SAU	SAU	SAU	SAU	-	-
A12	SAU	SAU	SAU	SAU	-	SAU*
A14	PMA	PMA	PMA	PMA	-	-
P1	SAU	SAU	SAU	SAU	-	-
P2	SAU	SAU	SAU	SAU	-	-
P4	PMA	PMA	PMA	PMA	-	-
	SAU	SAU	SAU	SAU	-	-
P7	SAU	SAU	SAU	SAU	-	-
P8	SAU	SAU	SAU	SAU	SAU*	-
		PAE		PAE		
P9	PMA	PMA	PMA	PMA	-	-
P12	SAU	SAU	SAU	SAU	SAU*	-
P14	AXY	AXY	AXY	AXY	-	-
	SAU	SAU	SAU	SAU	-	-
P18	SAU	SAU	SAU	SAU	-	-
P21	SAU	SAU	SAU	SAU	SAU	SAU
P29	SAU	SAU	SAU	SAU	-	-
	-	SMA*	SMA*	SMA*	SMA*	SMA*
P30	SAU	SAU	SAU	SAU	SAU*	-
P34	SAU	SAU	SAU	SAU	-	-
P35	AXY	AXY	AXY	AXY	-	-

	Sputum			Saliva			Toothbrush		
Patient	1st	2nd	3rd	1st	2nd	3rd	1st	2nd	3rd
P3	AXY	AXY	AXY	AXY	AXY	AXY	-	-	-
P17	PAE	PAE	PAE	PAE	PAE	PAE	PAE*	-	-
	SAU	SAU	SAU	SAU	SAU	SAU	SAU	SAU	SAU
	-	CAL	CAL	CAL*	CAL	CAL	-	CAL	CAL
P33	SAU	SAU	SAU	SAU	SAU	SAU	-	SAU*	-

### Presence of clonal strains in saliva/toothbrush and sputum

**Figure 2.**
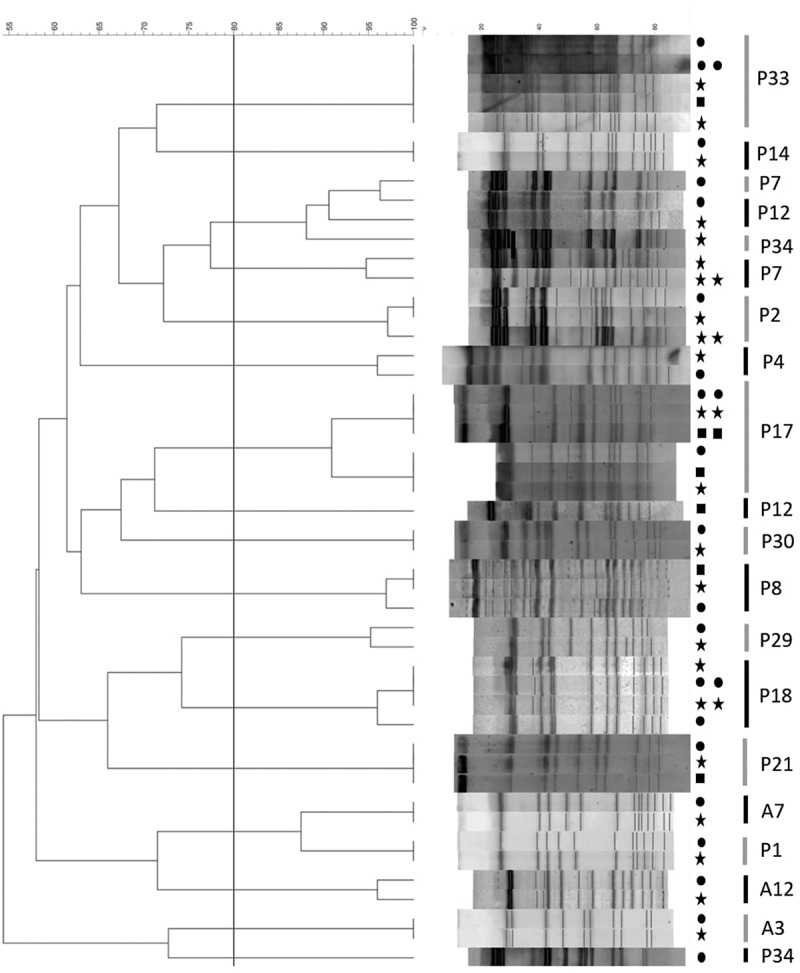
Phylogenetic trees obtained from the analysis of PFGE profiles of *S. aureus*. ● Sputum, Saliva and ■ Toothbrush. Two symbols in a row = two isolates. 80% similarity is indicated by the dashed line.

**Figure 3.**
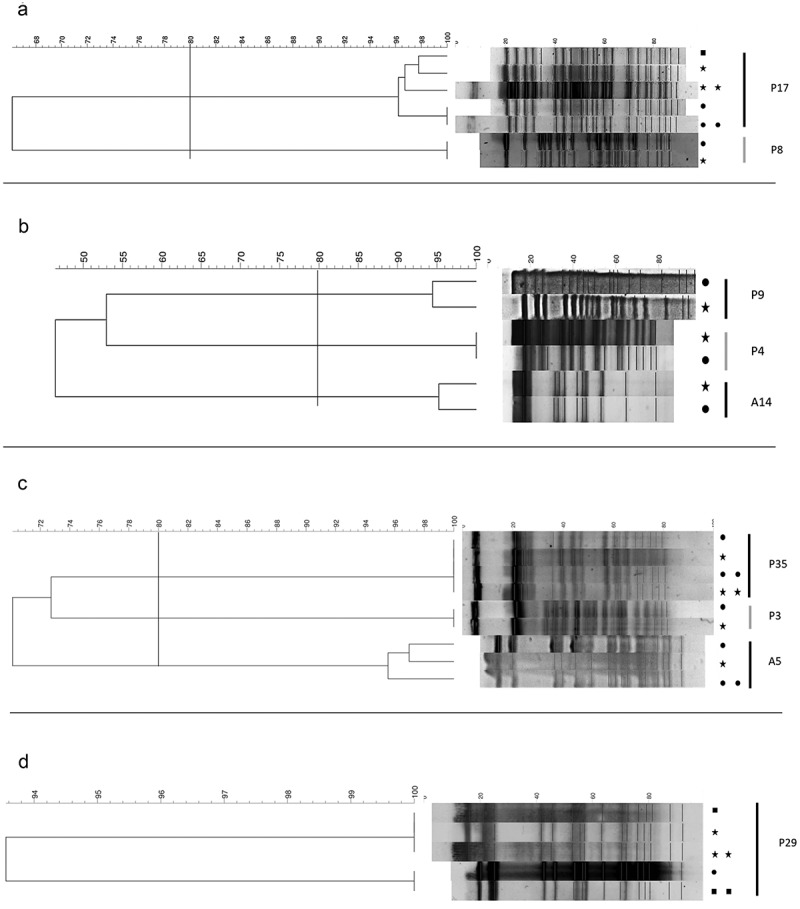
Phylogenetic trees obtained from the analysis of PFGE profiles of (**A**) *P. aeruginosa*, (**B**) *S. maltophilia*, (**C**) *A. xylosoxidans*, (**D**) *S. marcescens*. The dendrogram was established with PFGE profiles by similarity analysis by the Dice coefficient and the UPGMA. A percent genetic similarity scale is shown over the dendrogram. Each column represents the isolated species of the patient in a specified sample, and the percent genetic similarity between the isolated strains. ● Sputum, Saliva and ■ Toothbrush. Two symbols in a row = two isolates. 80% similarity is indicated by the line.

To verify the occurrence of transmission between the oral environment and lower airways, the clonal identity of the investigated species (*P. aeruginosa, S. aureus, S. maltophilia*, A. *xylosoxidans, S. marcescens*) isolated from sputum, saliva and/or toothbrush of the same patients was assessed by molecular typing (PFGE). According to the analysis of PFGE profiles of all the analyzed isolates, clonal identity (similarity ≥ 80%) of the strains among the different sites was confirmed in all cases with the only exception of two patients (P12, P34) presenting two clones of *S. aureus* among the different types of sample. Phylogenetic trees obtained from the analysis of PFGE profiles are shown in Figures 2 and 3.

## Discussion

The toothbrush is the main tool to keep the oral cavity and teeth clean in order to prevent dental problems such as caries, gingivitis, periodontitis, halitosis and aggravations of systemic diseases related to the oral cavity [26]. However, when bacteria survive on the toothbrush’s bristles, they may re-inoculate the oral cavity of the original host [16], thus increasing the risk of infectious diseases [27]. The toothbrush can be contaminated with microorganisms coming from both the oral cavity and the environment in which it is stored. The wet environment of the bathroom, as well as aerosols from toilet drains and contaminated hands, can contribute to the colonization of the toothbrush. In addition, most families store the toothbrushes in common containers leading to the possibility of cross-infection [26].

Microorganisms can first infect the oral cavity and then spread to the rest of the body causing serious health problems (e.g. heart disease, gastrointestinal, respiratory and kidney disease, as well as heart attacks, arthritis, etc.) or exacerbate existing diseases [27,28]. This can be particularly relevant in subjects at risk such as CF patients. The actual impact of the contaminated toothbrush on bronchial colonization in these subjects is difficult to assess since bronchial colonization can come from different sources and is highly dependent on the lung condition of the patients.

In this study, we aimed to evaluate the presence of typical and emerging CF bacterial species, namely *P. aeruginosa, S. aureus, S. maltophilia*, A. *xylosoxidans* and *S. marcescens* in saliva and in toothbrushes of CF patients, both adults and pediatrics. Our results indicated the presence of the investigated species in about 16% of the toothbrushes. These are comparable with data from Genevois and colleagues who detected *S. aureus* in 22% of analyzed toothbrushes [17]. As regards the saliva, a consistent presence of potentially pathogenic microorganisms was observed. The association between oral conditions and several respiratory conditions has been previously noted [29], and typical respiratory pathogens have shown to colonize the dental plaque of hospitalized intensive care and nursing home patients. Once established in the mouth, these pathogens may reach the lungs and cause pulmonary infection. The high percentage of potentially pathogenic microorganisms found in the saliva of the enrolled CF patients might be explained by recent findings on the saliva composition of CF patients. A significant decrease in the total, free and combined concentration of sialic acid in the saliva of CF patients compared to healthy subjects was identified [30]. Moreover, a decreased functioning of the salivary peroxidase system was observed in CF patients, resulting in lower levels of thiocyanates, which elicit antibacterial, antiviral and antifungal properties [31].

Regarding the different prevalences of the investigated species, the low percentage of *P. aeruginosa* was expected, since the patients were selected among those never or occasionally infected by this pathogen. The high prevalence of *S. aureus* was also expected, this species being the most frequent pathogen infecting pediatric patients. It is interesting to note that two bacterial species considered as emerging potential pathogens in CF and still lacking a clear clinical role, *A. xylosoxidans* and *S. maltophilia*, were found in a significant percentage in the samples examined in this study. Interestingly, among other potential pathogens, *Candida* spp. were isolated with high frequency from both saliva and toothbrushes. Furthermore, *Candida* spp. were the second most frequently isolated microorganisms from the three types of samples. These data highlighted the possibility that *Candida* spp. could have a pathogenic role in CF, as supported also by previous studies suggesting that *Candida* spp. are related to worsening of CF lung disease. However, currently there is not enough evidence to suggest that *Candida* spp. are responsible for a series of events including infectious exacerbations.

To investigate the possibility of transmission between the lower airways and oral environment, we assessed the contemporary presence of the bacterial species in saliva/toothbrushes and sputum samples of the same patients and checked their clonal identity by PFGE analysis. Clonal identity was confirmed in almost all cases, supporting the occurrence of transmission along the airways. Particularly, the case of patient P29 illustrated well the possibility of transmission of bacteria from the toothbrush to lower airways: at the first sampling, toothbrush and saliva of this patient were positive for the presence of *S. marcescens*, while the sputum sample was only positive for *S. aureus*. At the second sampling, sputum was also positive for the presence of the same *S. marcescens* clone previously isolated from saliva and toothbrush, highly suggesting a passage of bacteria from the environment/mouth to the lung.

Therefore, we can conclude that the toothbrush, an indispensable tool for daily oral hygiene, may be an important source of oral contamination and might act as a bacterial reservoir and favor transmission of potentially pathogenic microorganisms from the environment to the oral cavity, and eventually to the lower airways. This raises important issues, as CF patients mainly focus their attention on the main disease, sometimes overshadowing oral hygiene.

In order to prevent the toothbrush from becoming a receptacle of potentially pathogenic microorganisms, it is recommended to change it frequently, at least every 3 months or even more often for the most vulnerable subjects [32]. Since the frequent change of the toothbrush increases the cost of maintaining oral hygiene, a cheaper alternative might be its decontamination. Although there are little data in the literature regarding toothbrush sanitizing, one study indicates that soaking the toothbrush in 3% hydrogen peroxide or Listerine mouthwash greatly reduces (i.e. 85%) the bacterial load [33] while 20 min immersion in a disinfectant every 3 days did not eliminate the microorganisms present on the toothbrush [34]. Recent studies demonstrated that chlorhexidine had greater efficacy against the microorganism colonization/biofilms on the toothbrush’s bristles and exhibited inhibition of microbial growth [35]. Toothbrush sanitizer devices are available. Therefore, we encourage the development of oral hygiene protocols to be shared with CF patients as part of their daily routine in order to expand their hygiene education and decrease the chance of lung infection.
